# Comparative Studies on The Efficiency of Neem Leaves *Azadirachta indica* and Flubendazole Treatment Against *Diplectanum* in Sea Bass *Dicentrarchus labrax*

**DOI:** 10.1007/s11686-022-00544-2

**Published:** 2022-04-15

**Authors:** Salah M. Aly, Sayed N. Abou El-gheit, Habiba M. Essam El-Din

**Affiliations:** 1 Department of Pathology, College of Veterinary Medicine, Suez Canal University, Ismailia, Egypt; 2 Laboratory of Fish Diseases, Aquaculture Division, National Institute of Oceanography and Fisheries, NIOF, Cairo, Egypt; 3 Department of Aquaculture Diseases Control, Fish Farming and Technology Institute, Suez Canal University, Ismailia, Egypt

**Keywords:** Monogenea, Flubendazole, Neem leaves, Immersion treatment

## Abstract

**Purpose:**

*Diplectanum* is a life-threatening metazoan infecting the gills of Sea bass *Dicentrarchus labrax* causing a wide-ranging extensive economic loss in the aquaculture sector. This study has focused on verifying the most effective non-toxic dose of the Neem (*Azadirachta indica*) and (flubendazole) bath treatment on infested *D. labrax* fingerlings.

**Methods:**

In the first phase of the experiment, a total of 180 apparently healthy fingerlings were subdivided into six groups for each treatment. The tested concentrations were 0, 50, 100, 150, 200, and 250 mg L^−1^ for *A. indica* and 0, 10, 20, 30, 40, and 50 mg L^−1^ for flubendazole. The second phase was conducted for one week in five groups for each treatment. The first group was untreated healthy. The remaining groups were infested and received different concentrations of 0, 50, 100, and 150 mg L^−1^ & 0, 10, 20, and 30 mg L^−1^ for *A. indica* and flubendazole, respectively.

**Results:**

The most toxic dose exhibited high mortality rates at 200 & 250 and 40 & 50 mg L^−1^ for *A. indica* and flubendazole, respectively. In the second phase of the experiment, the most effective dose was 150 and 30 mg L^−1^; for *A. indica* and flubendazole, respectively. They demonstrated the lowest mortality rates 20.00 & 20.00 %, prevalence rates 43.33 & 23.33%, and mean parasitic intensities were 2.35 & 2.00 accompanied by the highest therapeutic efficacy value 67.85 & 74.6% for both treatments; respectively.

**Conclusion:**

The most effective anthelmintic efficacy has been assigned for flubendazole and *A. indica* at 30 and 150 mg L^−1^.

## Introduction

European Sea bass *Dicentrarchus labrax* is considered one of the most high-ranking profitable fish in Europe and the Mediterranean areas. Monogenetic trematodes are a frequent parasitic problem that causes significant worldwide economic losses [1, 2]*.* Monogenea is a subclass of Platyhelminthes which includes two main groups; *Polyopisthocotylea* and *Monopisthocotylea* comprising of *Dactylogyrus*, *Gyrodactylus*, *Furnestinia*, *Diplectanum *species [3].

Medicinal plants have been registered as having antimicrobial, immunostimulant, appetite stimulation, anti‐inflammatory, and antiparasitic properties [4]. They contain numerous bioactive compounds, such as alkaloids and glycosides, substitutes for natural parasitic control [5].

One of the most promising medicinal plants is the Neem *Azadirachta indica*, a broad pharmacological and environmentally friendly compound [6, 7]. The proficiency of *A. indica *has been successfully assessed to control caligid copepod infestation on Asian Sea bass (*Lates calcarifer*) [8]. freshwater (*Argulus bengalensis)* [7]**, **and the overpopulation of copepods in cultured Nile tilapia (*Oreochromis niloticus*), and African catfish (*Clarias gariepinus)* [9].

Flubendazole was chosen to compare the efficiency of herbal parasitic treatment versus chemical treatment measures. Flubendazole, mebendazole, fenbendazole, and oxibendazole (10 mg L^−1^) were proved to reduce the infestation level of *Pseudodactylogyrus* spp. [10]. In fish, flubendazole is used for controlling hydra, intestinal parasites (*Hexamita*, gill flukes, and *Camallanus*) mainly by adsorption through the fish’s skin. The used dosage of 10% flubendazole is 0.5 g per 20 gallons (75 L) reported to be safe without any delayed expression of toxicity for 21 days after a 96-h exposure [11].

Also, the gill worms or intestinal parasites have been treated using 340 mg of 5% flubendazole dissolved in 230 mg of dimethyl sulfoxide solution in 500 l of aquarium or pond water [12].

Therefore, this study aimed to estimate the toxicity range of neem leaves aqueous extract and flubendazole suspension in *D. labrax*. Furthermore, the effectiveness of treatment will be determined by calculating the prevalence rates, mean parasitic intensities, and therapeutic efficacy.

## Materials and Methods

### Fish Source

A total number of 220 (140 apparently healthy and 80 infested *D. labrax* 15 ± 0.7 g) fingerlings stock were obtained from Wadi-el-Rayan farm, Fayoum Governorate. The handling and examination of fish are based on the guidelines for fishes in the research described by [13]. Gill biopsies were obtained after 0.025% clove oil anesthesia [14], and then examined freshly under Stereo Microscope (Optika) [15]. The parasites were identified as *Diplectanum* species by the diagnostic outlines described by [16–18].

### Fish Acclimation

Fish were acclimatized for 1 week in fiberglass rearing tanks (one for healthy fish and the other for the infested ones). They were supplied fresh seawater, aeration, and a filtration system. The water parameters ranges were optimized at temperature 27.2 ± 0.96 °C, pH 7.3 ± 0.32, dissolved oxygen 7.12 ± 1.17 mg L^−1^, and 16 ± 0.69‰ salinity with a daily water exchange at 30%. Water quality was monitored following the protocol provided by [19]. Fish were fed twice daily at 3–5% of their body weight with a pelleted commercial ration containing 40% crude protein.

## Preparation of Stock Solutions of Treatments:

### Neem (*Azadirachta indica*) Aqueous Extract

Freshly harvested neem leaves were obtained from the Agriculture Research Center, Ministry of Agriculture, and Reclamation, Egypt. They were rinsed, dried, and crushed into powder form. A stock solution was obtained by infusion of 500 g/liter in distilled saline water at room temperature for 24 h, then filtered for any impurities, and used immediately at different concentrations [20].

### Flubendazole Suspension

It was acquired as Fluver® suspension 100 mg/5 ml (Alexandria Company for Pharmaceuticals and Chemical Industries).

### First phase: Toxicity Evaluation of Both *A. indica *and Flubendazole on Healthy *D. labrax*

For each treatment testing, six groups were assigned with ten fish per group, giving 120 fish per treatment. They were distributed in 20 L glass aquaria containing aerated seawater. Post-acclimatization, the different concentrations of *A. indica* and flubendazole were introduced to the corresponding treatment tanks.

The toxicity of both herbal and chemotherapeutic substances against healthy *D. labrax* was ascertained by determining the survival rate after treatment by ascending concentrations compared with the control (untreated seawater only). *A. indica* toxicity was tested at 50, 100, 150, 200, and 250 mg L^−1^ for 12 h [7]. Similarly, flubendazole was also assessed on 10, 20, 30, 40, and 50 mg L^−1^ for 12 h [21].

All the tested fish groups were observed and regularly recorded for any behavioral changes and mortality at 3, 6, 9, and 12 h. No fish were fed during the treatments [22].

### Second phase: Assessment of the Effective Therapeutic Doses of *A. indica* and Flubendazole Bath

Based on the toxicity test results, the highest toxic doses were excluded (200 &250 and 40&50 mg L^−1^ for* A. Indica* and flubendazole, respectively). Therefore, the tested doses were chosen below these levels. After acclimatization, the confirmed infested fish with *Diplectanum* were allocated separately in two experimental stations. Five groups (N = 10) were distributed in 20 L glass aquaria for 7 successive days for each station. The first groups of each station, (A 1) & (F1), were negative control (untreated healthy group), and the 2nd group (A 2) & (F 2) were positive control (infested but kept untreated). The groups (A 3), (A 4), (A 5), (F 3), (F 4), and (F 5) were infested and received different concentrations ranging from 50, 100, and 150 mg L^−1^ for *A. Indica *& 10, 20, and 30 mg L^−1^ for flubendazole, respectively [7, 23–25].

The fish were monitored for abnormal behavioral and clinical signs throughout the experimental period. Dead and morbid fish were removed, and cumulative mortality was recorded. After the seventh day of the treatment, the fish from the control and medicated groups were sacrificed. In addition, parasites on the skin and gills were counted with a Stereomicroscope. The fish mortality rate, prevalence percentage, and the mean parasitic intensity were recorded following [26]. The therapeutic efficacy was calculated according to the formula described by [27].

## Results

### Toxicity Evaluation of *A. indica* and Flubendazole (12-h bath)

The survival rate of *D. labrax* was recorded after exposure to different concentrations ranging between 0 and 250 mg L^−1^ *A. Indica* for 12 h. bath (Table 1). The treated fish groups from 0 to 150 mg L^−1^ did not show any mortalities or abnormal signs. In contrast, the fish start to die at the ninth hour of exposure at the concentration of 200 mg L^−1^ (G 5).

**Table 1:** The survival rate of treated *D. labrax* fingerlings by variable concentrations of *A. indica* for a 12-h bath

Exposure time (hour)	Survival rate (%)of *A. indica* (mg L^−1^)					
	G1 0	G2 50	G3 100	G4 150	G5 200	G6 250
3	100	100	100	100	100	96.1
6	100	100	100	100	100	94.9
9	100	100	100	100	98.2	93.7
12	100	100	100	100	97.3	92.2

Concerning the survival rate of *D. labrax* for flubendazole*,* the highest safe rate was achieved in 30 mg L^−1^ even after 12 h. Therefore, Groups G5 and G6 were considered the highest toxic levels and excluded from further examined parameters (Table 2).

**Table 2:** The survival rate of treated *D. labrax* fingerlings by variable concentrations of flubendazole for a 12-h bath

Exposure time (hour)	Survival rate (%)of flubendazole (mg L^−1^)					
	G1 0	G2 10	G3 20	G4 30	G5 40	G6 50
3	100	100	100	100	63.33	43.33
6	100	100	100	100	50	30
9	100	100	100	100	23.33	13.33
12	100	100	100	100	13.33	7.33

### Assessment of Effective Therapeutic Doses of *A. indica* and Flubendazole Bath

Table 3 shows that the fish mortality rate, prevalence rate, mean parasitic intensity, and therapeutic efficacy were assessed to determine the most effective dose from the remaining groups.

**Table 3:** The effect of *A. indica* bath for 7 successive days in the infested *D. labrax* with *Diplectanum*

Treatments Concentration	(A 1)* -ve Control	(A 2) + ve Control	(A 3) 50 (mg L^−1^)	(A 4) 100 (mg L^−1^)	(A 5) 150 (mg L^−1^)
Fish mortality (%)	**–**	44.44	33.34	27.78	20.00
Prevalence (%)	**–**	90.00	66.70	50.00	43.33
Mean parasitic intensity	**–**	7.33	4.33	3.66	2.35
Therapeutic efficacy (%)	**–**	–	40.47	49.71	67.85

*Non-infested fish with neither mortality nor parasitic infestation

For *A. indica *treatment, the treated groups of (A 3) 50 mg L^−1^, (A 4) 100 mg L^−1^, and (A 5) 150 mg L^−1^ exhibited a mortality rate of 33.34, 27.78, and 20%, respectively compared to the positive control (A 2). Simultaneously, a declined prevalence rate was noticed; the lowest mortality was in (A 5) by 43.33%. Moreover, lower mean parasitic intensities were noted in (A 3), (A 4), and (A 5) by 4.33, 3.66, and 2.35, respectively. Consequently, higher values of therapeutic efficacy 40.47, 49.71, and 67.85% were recorded in (A 3), (A 4), and (A 5), respectively.

Concerning the flubendazole, the treated groups (F 2), (F 3), and (F 4) with 10, 20, and 30 mg L^−1^ revealed a considerable decrease in mortality, prevalence rate, and mean intensity, and the lowest was in (F 5). Meantime the higher therapeutic efficacies, 64.28, 70.63, and 74.60%, were achieved in 10, 20, and 30 mg L^−1^, respectively (Table 4).

**Table 4:** The effect of flubendazole bath for 7 successive days in the infested *D. labrax* with *Diplectanum*

Treatments concentration	(F 1)* -ve Control	(F 2) + ve Control	(F 3) 10 (mgL^−1^)	(F 4) 20 (mgL^−1^)	(F 5) 30 (mgL^−1^)
Fish mortality (%)	**–**	42.22	18.89	21.11	20.00
Prevalence (%)	**–**	86.66	40	33.34	23.33
Mean parasitic intensity	**–**	6.68	2.33	2.00	2.00
Therapeutic efficacy (%)	**–**	**–**	64.28	70.63	74.60

*Non-infested fish with neither mortality nor parasitic infestation

In conclusion, the most effective dose was 150 and 30 mg L^−1^ for *A. indica *and flubendazole, respectively. These concentrations showed the lowest mortality, prevalence rates, and mean parasitic intensities accompanied by the highest therapeutic efficacy value.

## Discussion

The current study compares the anthelmintic efficacy of *Azadirachta indica* aqueous solution and flubendazole suspension treatment against *Diplectanum* infesting *D. labrax.* On the toxicity level, the *Azadirachta indica* typically as a herbal extract showed the highest survival rate with 92.2% even at the uppermost concentration at 250 mg L^−1^ compared to flubendazole which revealed the lowest rate with 7.33% at 50 mg L^−1^ after 12 h bath for both treatments. On the level of the therapeutic evaluation, after a 1-week treatment by flubendazole bath at 20–40 mg L^−1^ daily, high therapeutic efficacies were achieved (67.85% at 40 mg L^−1^). Flubendazole at (10 mg L^−1^) has reduced the infestation level of *Pseudodactylogyrus* spp, but it did not completely deactivate the parasite [10]. On the other hand, *A. indica* aqueous extract showed diminished fish mortality, prevalence, mean parasitic intensity rates, and higher therapeutic efficacy at a concentration of 150 mg L^−1^. A nearly similar result was obtained against *Argulus bengalensis* at 120–250 ppm of the aqueous solution of* A. indica* [7]. Furthermore, one of the most important factors in evaluating any veterinary treatment is the economic aspect, but it is difficult to define it accurately, because it varies from place to place and prices differ from one country to another, and depend on the availability of products, etc. [28]. In this study, the economic impact on the fish farmer was demonstrated by revealing the effect of both treatments on improving the fish mortality rates. The positive control groups in infested nontreated fish have reached approximately 42–44.5%. Flubendazole showed the lowest mortality rate with 18.89% at (10 mg L^−1^). However, both treatments demonstrated the same mortality rate with 20% at 150 mg L^−1^ and 30 mg L^−1^ for *A. indica* and flubendazole, respectively. An additional evaluation parameter is the applicability of both treatments. *A. indica* was considered the hardest, because it needed more preparation, storage, and calculation of concentration steps than flubendazole due to the lack of commercially available form. This made *A. indica* more difficult in the application and required more skilled laborers. Finally, it is concluded that flubendazole has been proven to give significant anthelmintic efficacy against *Diplectanum* infestation with a reasonable safety margin. Moreover, it revealed a minimal mortality rate, and also, it is a worldwide commercially available drug.

## Conclusions

Using *A. indica* aqueous extract with a concentration of 150 mg L^−1^ or flubendazole at the concentration of 30 mg L^−1^ bath treatment for 7 successive days was proven to manage the *Diplectanum* infestation in *D. labrax* without showing any toxic effects on affected fish.
